# Polystyrene-b-Poly(2-(Methoxyethoxy)ethyl Methacrylate) Polymerization by Different Controlled Polymerization Mechanisms

**DOI:** 10.3390/polym13203505

**Published:** 2021-10-12

**Authors:** Dragutin Nedeljkovic

**Affiliations:** College of Engineering and Technology, American University of the Middle East, Egaila 54200, Kuwait; Dragutin.Nedeljkovic@aum.edu.kw; Tel.: +956-2225-1400 (ext. 2196)

**Keywords:** block-copolymer, anionic polymerization, atomic transfer radical polymerization, macroinitiator, controlled polymerization, functional polymer

## Abstract

Functional polymers have been an important field of research in recent years. With the development of the controlled polymerization methods, block-copolymers of defined structures and properties could be obtained. In this paper, the possibility of the synthesis of the functional block-copolymer polystyrene-b-poly(2-(methoxyethoxy)ethyl methacrylate) was tested. The target was to prepare the polymer of the number average molecular weight (Mn) of approximately 120 that would contain 20–40% of poly(2-(methoxyethoxy)ethyl methacrylate) by mass and in which the polymer phases would be separated. The polymerization reactions were performed by three different mechanisms for the controlled polymerization—sequential anionic polymerization, atomic transfer radical polymerization and the combination of those two methods. In sequential anionic polymerization and in atomic transfer radical polymerization block-copolymers of the desired composition were obtained but with the Mn significantly lower than desired (up to 30). The polymerization of the block-copolymers of the higher Mn was unsuccessful, and the possible mechanisms for the unwanted side reactions are discussed. It is also concluded that combination of sequential anionic polymerization and atomic transfer radical polymerization is not suitable for this system as polystyrene macroinitiator cannot initiate the polymerization of poly(2-(methoxyethoxy)ethyl methacrylate).

## 1. Introduction

**Figure 1:**
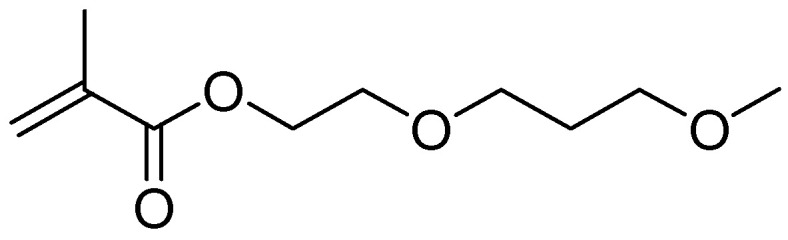
Structure formula of 2-(methoxyethoxy)ethyl methacrylate (DMEEMA).

Rapid change in solubility is the property of certain polymeric materials that can be utilized for the construction of the novel membranes. This type of membranes would change their permeability as a response to the temperature changes. Those membranes could be applied as the temperature-triggered controller for the separation of proteins and/or controlled drug delivery [1,2]. Therefore, great efforts were directed towards the synthesis of temperature-sensitive polymers which are suitable for the construction of the mentioned membrane [3]. As the main application of membrane should be around 37 °C (normal temperature of a healthy human body), polymeric material used for this purpose must be chemically and mechanically stable, non-toxic, non-biodegradable with a switching temperature below 40 °C (otherwise the proteins would degrade at the switching temperature) [4]. Possible approach to the solution may be diblock-copolymer in which one block shows lower temperature solution temperature (LCST) behavior while the other block has high mechanical stability and serves as a mechanical carrier [5,6]. The blocks of synthesized diblock-copolymer will be polystyrene (PS) (as the mechanical carrier) and poly(2-(methoxyethoxy)ethyl methacrylate) (*PDMEEMA*) (as the active component). DMEEMA shows LCST point at 26 °C [7,8,9]. The formula of the DMEEMA monomer is presented in Figure 1.

This system has been chosen as block-copolymerization of similar (but different) polystyrene-b-poly(methylmethacrylates) has been well described and behavior of the methacrylate monomers is known in similar polymerization systems [10]. It is assumed that DMEEMA, despite having long and polar side chains, would behave in an analogous way as methacrylates with shorter side chains [11]. Although similarities in polymerization behavior between DMEEMA and other methacrylates are expected, attempts to synthesize block-copolymer containing DMEEMA in a controlled manner have not been attempted so far. Synthesis of block-copolymers with solubility switching temperature in the desired range (e.g., polystyrene-b-polyacrylamide) have been performed by the free radical mechanism [12,13]. However, use of the free radical mechanism causes difficulties in control of the molecular mass and the differences in the lengths of the chains of synthesized polymer. Broader mass distribution may negatively affect phase separation, so a system suitable for controlled polymerization was chosen.

In order to utilize LCST behavior, phase separation must occur with component with lower fraction (*PDMEEMA*) cylinders packed in the PS matrix. This particular structure would provide pore openings through the entire active layer of the membrane [14]. This structure can be obtained if the fraction of the minor component (in this case PDEEMA) is between 0.2 and 0.4 (by mole) [15,16,17]. Dispersity must be as low as possible in order to preserve microphase separation. Finally, synthesized diblock-copolymer should have high number average molecular weight (Mn) (at least 100) for the adequate mechanical stability. All those challenges could be fulfilled using different mechanisms for the controlled polymerization. In this work, the polymers were synthesized using sequential anionic polymerization (SAP), atomic transfer radical polymerization (ATRP) and the combination of SAP and ATRP.

The SAP is well known as a suitable mechanism for the synthesis of the block-copolymers with defined structures and the lengths of the different blocks. It is widely used for the polymerizations of the monomers that contain electron accepting substituents (methacrylates among the other monomers) [18]. Obtained polymers typically have narrow molecular weight distribution, with typical values for dispersity of 1.15–1.2. This reaction occurs between carbanion on the living chain end of propagating chain (accompanied by the counter-ion) and the monomer. As the termination does not normally occur in the anionic polymerization, every “living” chain end behaves as the macroinitiator. Therefore, when the monomer is consumed, addition of a new batch of different monomers would yield a diblock-copolymer. However, as the living carbanions are extremely sensitive to the presence of any electrophilic species, the setup for the SAP is complex, and the procedure of the preparation of all required chemicals is tedious and demanding [19,20].

Atomic Transfer Radical Polymerization (ATRP) is type of controlled (“living”) polymerization based on a transfer of the atom to the radical. It is one of the most versatile mechanisms, which can be applied to the wide range of monomers and solvents [21,22]. Contrary to the comparative systems for controlled polymerization, it can be performed under various conditions, even in water as the solvent [23]. Successful polymerizations of methacrylate monomers have been reported by this system [24,25]. Another advantage of the ATRP is that it can be initiated by the polymers synthesized by different mechanisms if they contain appropriate end groups [26]. This is especially important because this way it is possible to obtain defined block-copolymers [27]. The main disadvantage of the ATRP mechanism for synthesis of block-copolymers is that obtained macroinitiator must be purified and dried before being used in the subsequent reaction. ATRP mechanism is tolerant to small amounts of oxygen in the system and typically yields the polymers with the polydispersity of around 1.4 [28,29]. Therefore, ATRP was chosen as one of the synthesis mechanisms for the experiments conducted in this work.

As the LCST point of *PDMEEMA* is close to the room temperature, this polymer system would potentially be applicable for any separation process based on the porous membranes that is performed at the conditions near room temperature. Block-copolymer should combine good mechanical properties of PS and changing solubility of *PDMEEMA*.

## 2. Materials and Methods

The synthesis of the diblock-copolymer PS-b-*PDMEEMA* was attempted by three different controlled polymerization mechanisms: SAP; ATRP and the combination of both. All reactants, catalysts and solvents were supplied by Sigma Aldrich and used as received, unless stated otherwise in text. Experimental procedures and the reaction schemes for each of the syntheses were as follows:

### 2.1. Sequential Anionic Polymerization

Anionic polymerization has been known for its great sensitivity to any kind of oxidative or protonating species. Therefore, reactants, solvents and the equipment that was used had to be kept under absolutely dry and oxygen-free conditions. Reactor for polymerization was made of double wall glass, with the volume of 2 dm^3^. It was cooled by a silicon based cooling liquid circulating between the walls. The first step in cleaning was by washing with methanol and tetrahydrofurane (THF). The cleaned and empty reactor was flushed with excess of nitrogen and kept under high vacuum. This procedure was performed twice, so any residual methanol was removed. Potential gaseous contaminants (mainly oxygen and to certain extent carbon dioxide) were removed from the reactor by the high vacuum line. Two cooling traps with liquid nitrogen were positioned between the reactor and the pump, in order to prevent any chemicals of reaching pump. Transfer of all chemicals used (solvent and monomers) to the reactor was done through vacuum line by applying under- or overpressure. Overpressure was created by pure and dry nitrogen. Transfer of initiators and additives to reactor was done using conventional syringe techniques (nitrogen counter stream was applied). Flushing of all syringes with argon or nitrogen was done prior to use. By this method of transfer, entering of moisture and oxygen was prevented as much as possible. Solvents (THF and toluene) were distilled in two step distillation process (with the reflux over potassium) and directly transferred into the reactor. Prior to polymerization or distillation, glassware was dried at 70 °C and then heated under vacuum up to 650 °C. The purpose of this procedure was to remove moisture as much as possible. Commercial DMEEMA which normally contains stabilizers which must be removed was treated by passing through an aluminum oxide column. Activated *PDMEEMA* was flushed with dry argon, treated by three freeze-pump-thaw cycles and finally distilled under high vacuum. The same procedure was repeated for every polymerization attempted because of the property of pure *PDMEEMA* to polymerize at the temperatures as low as −18 °C when stored for a few hours. The activation of styrene was done by passing through an aluminum oxide column, distilling under reduced pressure and storing over CaH_2_. Similarly, *PDMEEMA* was degassed by three freeze-pump-thaw cycles and distilled prior to reaction. As the initiator, commercial sec-buthyl-lithium solution in cyclohexane was used. As PMEEMA carbanion cannot initiate the polymerization of styrene, the PS block was synthesized first.

**Figure 2:**
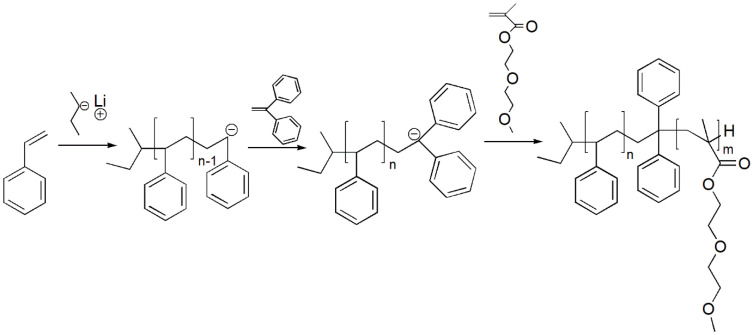
Reaction scheme of the synthesis of the PS-b-*PDMEEMA* block-copolymer by the SAP.

Solvent was transferred to the reactor and treated with 5–10 mL of sec-BuLi solution in cyclohexane (1.4 M) overnight. Styrene was introduced to the reactor and the reaction was typically running for two hours at the temperature of −63 °C. If methacrylate monomer is added directly to living chain end of polystyrene, two polymerization reactions are possible. Beside expected and desired propagation through the vinyl group, it is possible that propagation occurs through the carbonyl bond yielding the unwanted product. To avoid this reaction, the nucleophylicity of the living polystyrene chain end must be decreased. This is performed by the addition of 1,1-diphenylethene (DPE). When the DPE unit carries the negative charge of the carbanion, the propagation through the carbonyl bond is not possible due to the steric reasons. For the same reason, the homopolymerization of the DPE by the anionic mechanism is not possible. Therefore, DPE is added in excess. After addition of the DPE reaction proceeded for additional 30 min, every living chain end contained exactly one DPE unit. Commercial DPE was treated with BuLi solution in cyclohexane and then distilled under vacuum conditions. After this end-functionalization reaction, the DMEEMA was added and the reaction continued at the same temperature for additional two hours. Reaction was terminated with degassed methanol. Precipitation was done in methanol, and Polymer was dried on the vacuum at 40 °C overnight. Detailed scheme of the synthesis of the PS-b-*PDMEEMA* via SAP is presented in Figure 2:

### 2.2. Atomic Transfer Radical Polymerization

The ATRP reactions were performed in the nitrogen-filled glove box at room temperature. The reaction was performed in 200 mL flask in the anisole as the solvent. Commercial, *pro analysis* grade anisole was flushed with nitrogen and degassed by three freeze-pump-thaw cycles. Ethyl α-bromoisobutyrate (EiBuBr) was used as an initiator, copper (I) bromide (CuBr) as an additive and N,N,N’,N”,N” pentamethyldiethylenetriamine (PMDTA) as a ligand forming agent. The initiator (α-EiBuBr) was stirred over CaH_2_, degassed and distilled at reduced pressure prior to the reaction. The ligand forming agent (PMDTA) was flushed with nitrogen prior to the reaction. CuBr was dissolved in anisole and the monomer that forms the first block and the ligand forming agent were added. The reaction was performed for four hours, and it was terminated by exposing the reaction mixture to ambient air (and thus, oxygen). The formed Cu complex was removed in the column filled with Al_2_O_3_, and the polymer was precipitated in methanol and dried. The polymer obtained by this way contains a bromine group at the end and should serve as the macroinitiator for the subsequent polymerization of the second block.

**Figure 3:**
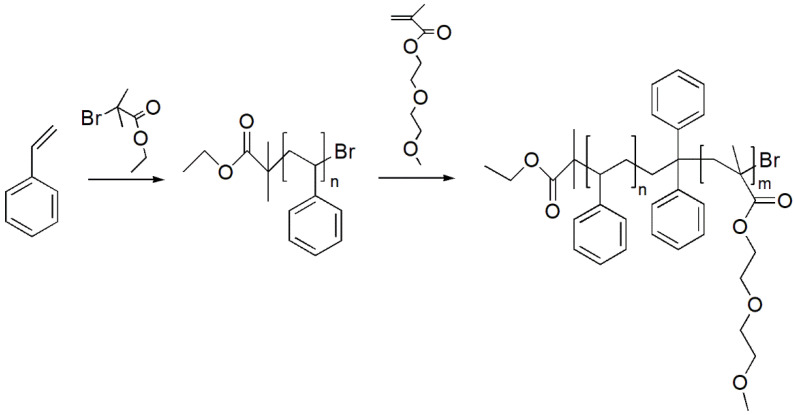
Reaction scheme of synthesis of the PS-b-*PDMEEMA* block-copolymer by ATRP.

The second (*PDMEEMA*) block was synthesized in an analogous reaction as the PS block. Initiator, additive and ligand forming agent were synthesized PS end-functionalized with bromine group, CuBr and PMDTA, respectively. Polymerization of the *PDMEEMA* block was performed in degassed anisole at the room temperature for four hours. Chemicals, including solvent, were prepared in the same way as in the case of the PS macroinitiator. Termination was done by exposing the mixture to ambient oxygen. Precipitation was performed in methanol, and the sample was dried in vacuum at 40 °C overnight. Reaction scheme of the ATRP synthesis procedure is presented in Figure 3:

### 2.3. Combination of the SAP and ATRP

**Figure 4:**
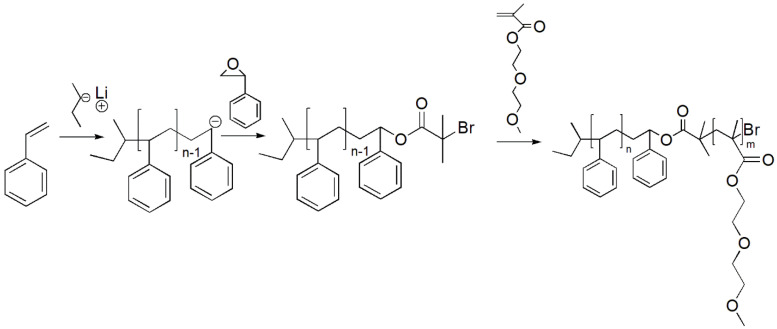
Reaction scheme of the synthesis of PS-b-*PDMEEMA* block-copolymer combination of SAP and ATRP.

In this approach, good properties of anionic polymerization (narrow molecular weight distribution, end-functionalization) were combined with ATRP (simpler procedure and experimental setup). The anionic synthesis of the PS macroinitiator was performed in THF solvent at −70 °C, initiated by the sec-BuLi, as described in the anionic polymerization procedure. After polymerization of the PS block in duration of two hours, precursor was taken (reaction terminated by the methanol). Before addition of the agent that should provide end-functionalization of the macroinitiator, styrene oxide (SO) was added. According to reports, the presence of the SO unit at the living chain-end of the propagating PS chain increases the probability of attaching the α-bromo-isobutyric group [30,31]. The end-functionalization with the SO proceeded at the same temperature for additional two hours. After this reaction, α-bromo-isobutyric acid bromide (α-BIAB) was added. The mixture was heated to the room temperature and stirred overnight. On the following day the reaction was terminated by adding the methanol, and the polymer was precipitated (in methanol) and dried overnight (in vacuum at 40 °C). Prior to polymerization of the second block, polymer was dried at the high vacuum line in order to remove any traces of moisture or residual methanol. ATRP synthesis of the second block was performed in the analogous way and under the same conditions as described in the ATRP section. Detailed reaction scheme for the synthesis of the PS-b-*PDMEEMA* by the combination of SAP and ATRP is presented in Figure 4:

### 2.4. Characterization

Molecular weights (both number and weight average) were determined by the Size Exclusion Chromatography (SEC) versus polystyrene calibration. Cross-linked polystyrene was used as the stationary phase. Tetrahydrofuran (THF) was used as a solvent, and the measurements were performed at the room temperature. and 2,6-di-tert-butyl-4-methylphenol as internal standard. VWR-Hitachi 2130 pump with the flow rate of 1.0 mL/min. The RI detector was a Waters 2410 (λ = 930 nm); the UV detector was a Waters, operated at 254 nm or 300 nm. Samples were injected using Waters 717 autosampler, with injection volumes of 20 μL. PSS WinGPC Unity software was used for data acquisition, correction and analysis.

The concentration was determined by simultaneous measurements by UV detector (at 254 nm which is the adsorption wavelength of the phenyl ring) and RI detector. The presence of β-keto ester was tested by UV measurement at 300 nm. The flow was 1 mL/min and the sampling volume was 20 µm. The aim of measuring both UV and RI signal is to check the presence of *PDMEEMA* block. As polystyrene shows both UV (254 nm) and RI signal, and *PDMEEMA* shows only RI signal, constant UV (254 nm)/RI value will imply absence of any *PDMEEMA* block. On the other hand, if UV (254 nm)/RI shows a slope, it may imply presence of both PS and *PDMEEMA* blocks.

Composition of copolymer samples was determined by the nuclear magnetic resonance (NMR). The solvent was deuterated chloroform (CDCl_3_), and the signal of chloroform (CHCl_3_) in CDCl_3_ (7.26 ppm) was used as a reference. All chemical shifts are presented versus tetramethylsilane. The NMR apparatus was Bruker AV300 with operating field of 7 T.

The composition of the copolymer was calculated by analyzing the NMR spectra as:

(1) $$x\left(PDMEEMA\right)=\frac{\frac{I\left(3-4.5\right)}{11}}{\frac{I\left(6-7.5\right)}{5}+\frac{I\left(3-4.5\right)}{11}}$$

In this equation *I*(*x*–*y*) represents the value of the integral in the range *x*–*y*. As all protons from DMEEMA units (11 protons altogether) have their chemical shifts in CDCl_3_ between 3 and 4.5 pm, numerator represents relative number of DMEEMA units. Denominator represents summation of number of DMEEMA units and styrene units (5 protons, all of them with chemical shifts between 6 and 7.5 ppm in CDCl_3_).

## 3. Results

The experimental results obtained for sequential anionic polymerization (SAP) are presented in Table 1.

**Figure 5:**
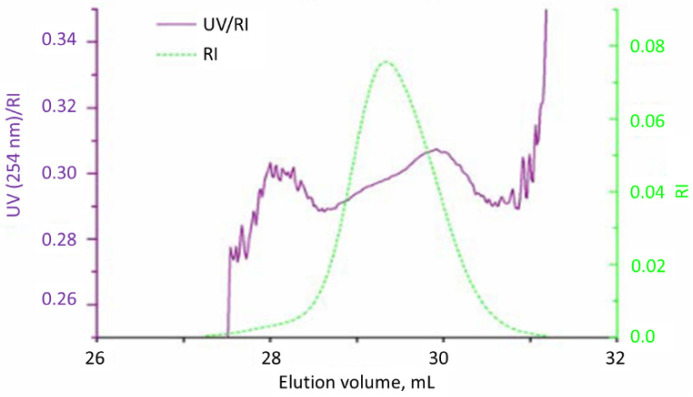
SEC elugram of Sample A1.

Molecular weights of all samples were determined by the SEC column (THF as a solvent) versus polystyrene calibration. As it can be observed from Table 1, samples with the lower Mn have shown good results. Sample A1–A4 is the fraction of DMEEMA in which the proper phase separation may be expected. The potential reason for significantly higher value of dispersity for Sample A4 is that the polymerization reaction did not proceed in the expected way. This hypothesis is (at least partly) corroborated by both apparent Mn and composition of the Sample A4 which significantly deviate from the theoretical calculation. Sample A3 contained more *PDMEEMA* than theoretically expected. A possible explanation for this result may be that some of the PS living chains were deactivated when the DMEEMA monomer was introduced in the reactor. The SEC elugram of Sample A1 is presented in Figure 5.

**Figure 6:**
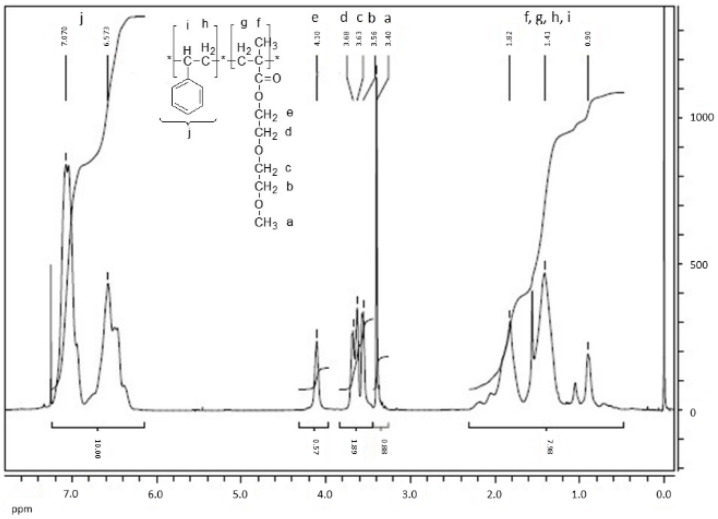
^1^H NMR spectrum of Sample A1 with the protons assigned to the appropriate chemical shifts.

As it can be seen, the monomodal distribution with narrow molecular weight dispersion was obtained (Đ = 1.05). In order to check the potential presence of the *PDMEEMA* block in this polymer, the ^1^H NMR was performed. The solvent was CDCl_3_ for all the samples. The result is presented in Figure 6. Proton assignments and the appropriate chemical shifts are also presented in Figure 6.

**Figure 7:**
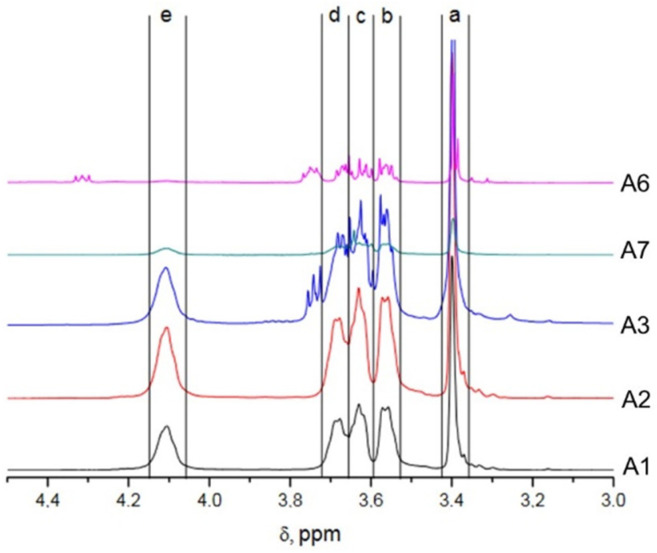
Comparison of NMR spectra of PS-b-*PDMEEMA* samples of different Mn.

Molar fraction of styrene is calculated by Equation 1 to be 87%, which is recalculated to the mass fraction of 78% for polystyrene. Those results are in accordance with the predicted calculated values, and similar results were obtained for samples with comparable number average molecular weights (A2–A4). Based on those results, polymerization of the same copolymer but with higher Mn (in the range 100–200) were attempted (Samples A5–A7). However, obtained copolymers did not contain a sufficient amount of DMEEMA (if any). The resulting NMR spectra with an emphasized part specific for the side chain of DMEEMA is presented on Figure 7. The H atoms assignment is the same as presented in Figure 6.

**Figure 8:**
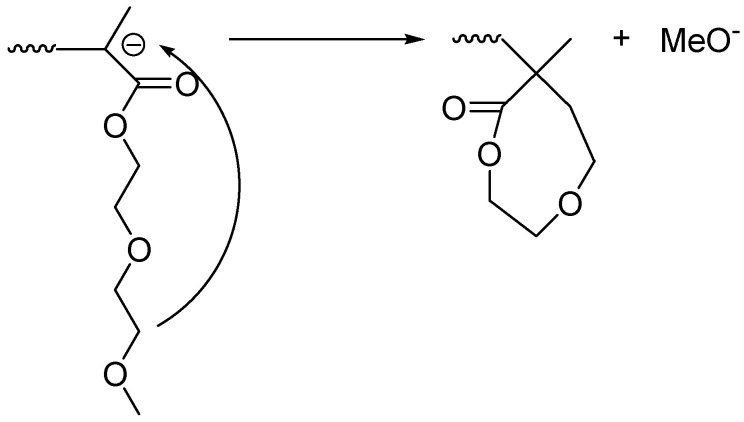
Potential termination of the chain propagation by the reaction of the living chain end with its own side-chain with the formation of 8-member ring.

**Figure 9:**
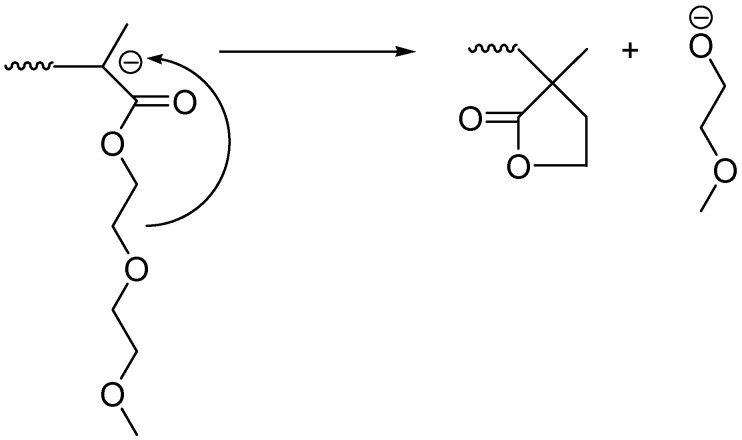
Potential termination of chain propagation by reaction of the living chain end with its own side-chain with the formation of 5-member ring.

As it can be seen from Figure 7, even with the bare eye it is obvious that the amount of DMEEMA incorporated in the copolymer decreases with the increased number average molecular weight. Comparing the detected amounts of DMEEMA in block-copolymer (Table 1), it is obvious that samples with lower molecular Mn (Samples A1–A4) contain amounts of DMEEMA that are in the expected range of the Mn calculated from the amounts of reactants taken. On the other hand, for higher Mn polymers (Samples A5–A7), only a few units of DMEEMA were detected (if any). A possible reason for the lack of DMEEMA block may be the occurrence of the unwanted side reactions. Well-known side reactions in anionic polymerization of methacrylates is reaction of living chain-end with the penultimate unit of already polymerized DMEEMA. If this reaction occurred, cyclic β-keto ester would be formed at the end of each chain terminating the propagation reaction. End group (β-keto ester) could be easily detected by its characteristic adsorption at 300 nm by UV detector [32]. However, UV measurements at 300 nm did not show any traces of β-keto ester. One of the possible side reactions is the reaction of the DMEEMA living carbanion with its own side chain. This reaction may be possible due to polarity of C-O bond in the side chain. This leaves the carbon atom with the partial positive charge that could react with its own living anion chain end. Schemes of those potential reactions are presented in Figure 8 and Figure 9:

Both of those reactions result in the dead chain end that effectively terminates the polymerization and the chain of *PDMEEMA* cannot propagate further. The chemical environment of the protons in the cyclic end of the chain would be relatively similar to the environment of the protons in the *PDMEEMA* chain which would explain the absence of the distinctive signal of the cyclic protons. In order to test the possibility of the formation of the cyclic esters at the end of the chain, the simulation of the ^1^H NMR was performed by MesteRec software package. Predicted spectra are presented in Figures S1 and S2.

Comparing the predicted signal (Figure 8 and Figure 9), it can be seen that potentially formed cyclic esters would have the distinctive signals at the chemical shifts of 4.2–4.3 ppm (for 8-member ring) and 4.3–4.4 (for 5-members ring). Other signals would be in the ranges of chemical shifts that are equal or very close to the shifts of *PDMEEMA*. If those predicted spectra are compared with those obtained and presented in Figure 7, it can be seen that the Sample A6 shows weak signal at the chemical shift of 4.3 ppm. This signal is sufficiently distinctive from other signals that are expected to be the result of *PDMEEMA* side chain hydrogen atoms. This signal also cannot come from the PS hydrogens or from the hydrogens from the main chain (see Figure 3 for their chemical shifts). As each chain can potentially contain only one cyclic end-group, this could also explain the low intensity of the signal. Based on comparison of the predicted and actual spectra, it can be concluded that the actual termination of the DMEEMA polymerization may occur by one of the paths presented in Figure 8 and Figure 9. However, due to the position and very low intensity of the signal, it is very hard to accurately judge by which of the two presented mechanisms the actual termination occurs.

Another possible reason for the termination is the presence of impurities in the DMEEMA monomer that is added to the reactor. Despite all the purification procedures and precautions, it is still possible that some of the moisture or oxygen enters the system. This cause of the termination is not so obvious in the samples with lower molecular weights as they contain a higher number of living chain ends in absolute terms.

As a second mechanism, the ATRP was performed. The amounts of chemicals taken and results are compiled in Table 2:

The labels in the table have the same meaning as described in Table 1. The SEC elugram of the Sample R1 is presented on Figure S3.

**Figure 10:**
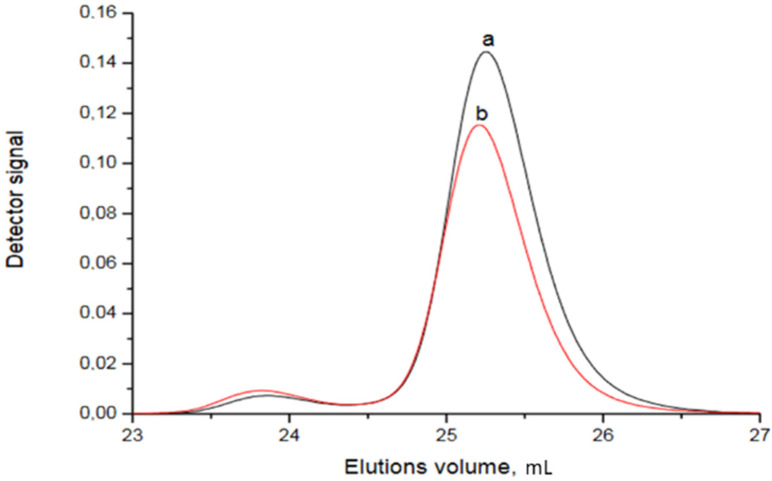
SEC elugram of Sample R2: (a) macroinitiator; (b) diblock-copolymer.

As it can be clearly observed from Figure S3, Sample R1 shows a bimodal distribution. The peak at elution volume of 29 mL corresponds to apparent number average molecular weight of 15.9 and the synthesized diblock-copolymer. The peak at the elution volume of 31 mL corresponds to residual PS macroinitiator. A relatively high fraction of residual PS indicates that a significant amount PS macroinitiator and lower Mn of synthesized block-copolymer clearly indicates that not all of the PS macroinitiator was involved in the reaction. However, as approximately 40% PS acted as the macroinitiator, synthesis of the Sample R2 with a higher Mn was attempted. The resulting elugram is presented in Figure 10:

Comparing the curves for the macroinitiator and for diblock-copolymer, it is obvious that no DMEEMA polymerized at all (this was confirmed by the NMR analysis). Small peak around 24 mL may correspond to the coupled chains of polystyrene. Obtained number average molecular weight is 154 with the dispersity of 1.06. Obtained results corroborates the results obtained by the anionic polymerization that PS-b-*PDMEEMA* of Mn around 20 can be obtained, while the same block-copolymer with Mn above 100 cannot. Almost perfect overlapping of curves in Figure 10 indicates that not a trace of *PDMEEMA* is present in Sample R2. Similarly, in the SAP case, only diblock-copolymer of relatively low molar mass could be obtained.

The next attempt was to synthesize PS based macroinitiator by anionic polymerization, end-functionalize it by the proper buthyl group and use it for ATRP of the DMEEMA. As in previous cases, polymerization of one sample with the lower (20–30) and one sample with the higher (above 120) Mn were attempted. The results are presented in Table 3.

**Figure 11:**
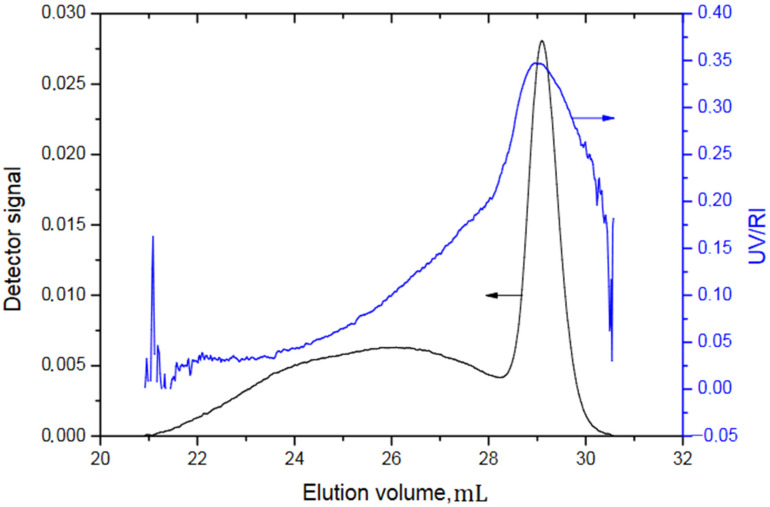
SEC elugram of Sample C1.

**Figure 12:**
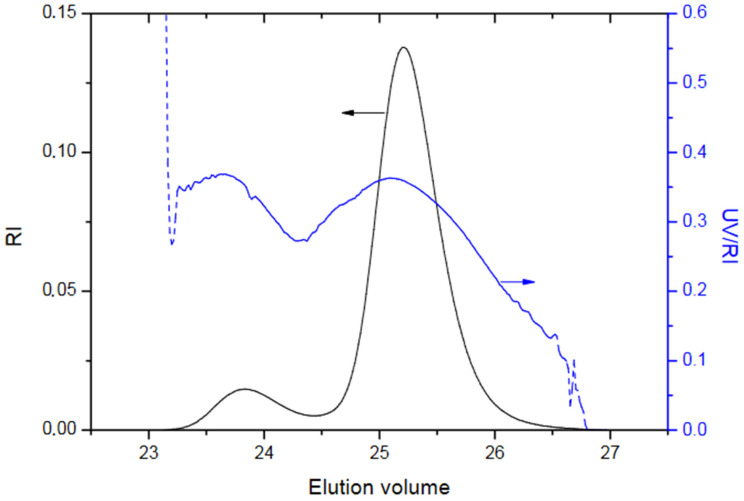
SEC elugram of Sample C2.

As it is obvious from Figure 11, the synthesis of the polystyrene macroinitiator was successfully performed (peak at 29 mL, corresponding to the apparent number average molecular weight of 31.3). However, the polymerization of DMEEMA in second stage was done in an uncontrolled way, yielding very broad distribution (broad signal between 21 and 28 mL) and relatively high Đ (1.6). This confirms expected results, as ATRP usually yields the polymer of higher polydispersity index in comparison to SAP. From the graph it is also obvious that only a fraction of the macroinitiator was effectively involved in the reaction and that most of the polystyrene did not get coupled with DMEEMA (either it was not capped with the bromine end group or it did not initiate the DMEEMA polymerization). However, as *PDMEEMA* was obtained up to the certain level, the synthesis of the block-copolymer with higher Mn by combination of SAP and ATRP was attempted (Sample C2). The result is presented in Figure 12.

SEC of the Sample C2 (Figure 12) shows bimodal distribution with peak at higher elution volume (25.5 mL) coming from the PS macroinitiator. This is confirmed by UV/IR ratio of 0.35 which is specific for polystyrene. As UV/IR ratio for the peak at the lower elution volume is approximately the same, it is reasonable to conclude that no DMEEMA polymerized by this mechanism and that this peak comes from coupled pairs of polystyrene that are formed during macroinitiator synthesis.

Based on the presented results, it may be concluded that, similarly to SAP and ATRP cases, PS-b-*PDMEEMA* of molecular weights of to 30–40 could be obtained. On the other hand, polystyrene macroinitiator that carries BIAB group with number average molecular weight of 160 could not initiate polymerization of DMEEMA by ATRP mechanism. Contrary to SAP case, no traces of DMEEMA were detected in high-molecular mass polymer. All three attempted mechanisms yielded a similar result. It was possible to obtain diblock-copolymer with molecular weight up to 40 while all attempts to synthesize block-copolymer that contains at least 20 wt % of *PDMEEMA* with molecular weight above 150 failed.

## 4. Discussion

The main goal of this work was to synthesize the block-copolymer PS-b-*PDMEEMA* that contains 20–40 mol% of DMEEMA for potential application in membrane separation technology. Syntheses of the polymers were performed by sequential anionic polymerization, atomic transfer radical polymerization and combination of the sequential anionic and atomic transfer radical polymerizations.

Comparing the results obtained by three different mechanisms, the general conclusion is that PS-b-DMEEMA can be synthesized by the SAP with the molar mass up to around 30 with PDI remaining low. Synthesis using the ATRP yielded the polymers of the comparable molar mass, but with higher values for PDI, while the combination of SAP and ATRP did not produce acceptable polymer. Synthesis of higher molar masses copolymers (>120) with any of the mechanisms was not successful. In the SAP mechanism, the reaction could not proceed beyond few units of DMEEMA being synthesized, and the most probable side reaction was reaction of the DMEEMA carbanion with its own side chain due to the partial positive charge on the carbon atoms in the side-chain. If this reaction occurs, indicated by the comparison of actual and predicted ^1^H NMR, the cyclic ester is formed at the end of the chain terminating the reaction after few units of DMEEMA are polymerized. Another possible reason for absence of the *PDMEEMA* block might be presence of the impurities in the DMEEMA monomer despite the careful and tedious preparation procedure. The termination of lower molecular weight polymers would not be observed because of the higher absolute number of living chain ends present in the system. In the case of the ATRP and combination of SAP and ATRP, no progress in the length of the chain was obtained between the blocks of higher molar masses. Therefore, it is reasonable to conclude that polystyrene macroinitiator is not capable of initiating the polymerization of DMEEMA. As the future challenge, the mechanical properties of the functional diblock-copolymers with the molar mass of approximately 30 will be tested for the potential application.

## Figures and Tables

**Table 1:** Results of SAP of PS-b-*PDMEEMA*.

Sample Number	n (S), mmol	n (DMEEMA), mmol	n (In), mmol	Mn (PS) (theo.)	Mn (D) (theo.)	Mn (SEC)	Mn (NMR)	Đ	x (DM), %
A1	104.4	18.9	0.42	25.9	8.5	18.8	22.1	1.05	13.2
A2	51	15	0.40	13.3	7	14	19.8	1.15	22
A3	43.5	10.9	0.21	21.5	9.8	16.3	27.1	1.17	36
A4	43.5	21.7	0.20	22.7	20.4	33.9	44	1.60	18
A5	43.5	21.7	0.05	90.7	81.7	90.2	90.3	1.12	0
A6	78.3	21.8	0.057	143	72	178.7	179.6	1.07	0
A7	87	37	0.066	137	107	284.4	293	1.09	2

The table columns are labeled as follows: n (S), mmol—number of moles of styrene monomer in mmol. n (DMEEMA), mmol—number of moles of DMEEMA monomer in mmol. n (In), mmol—number of moles of the initiator (sec-buthyl-lithium) in mmol. Mn (PS) (theo.)—theoretical expectation for the number average molecular weight of the PS blockl. Mn (D) (theo.)—theoretical expectation for the number average molecular weight of the *PDMEEMA* block. Mn (SEC)—number average molecular weight of PS block. Mn (NMR)—number average molecular weight of the diblock-copolymer (apparent molecular weight versus polystyrene calibration obtained by the SEC). Đ—dispersity, calculated as Mw/Mn (both of molecular weights are apparent versus polystyrene calibration). x (DM)—molar fraction of the DMEEMA block, obtained from the ^1^H NMR spectroscopy.

**Table 2:** Results of the ATRP of PS-b-*PDMEEMA*.

Sample Number	n (S), mmol	n (DMEEMA), mmol	n (In), mmol	M (PS) (theo.)	M (DM) (theo.)	Mn (SEC)	Mn (NMR)	Đ	x (DM), %
R1	43.5	8	0.02	27	7.5	11.5	15.9	1.21	23.1
R2	97	8	0.02	135	30	153	154	1.06	0

**Table 3:** Results of the synthesis of PS-b-*PDMEEMA* by combined SAP-ATRP mechanism.

Sample Number	n (S), mmol	n (DMEEMA), mmol	n (In), mmol	M (PS) (theo.)	M (DM) (theo.)	Mn (SEC)	Mn (NMR)	Đ	x (DM), %
C1	87	5.4	0.05	18.5	20.3	30.6	36	1.6	11
C2	52	5.4	0.007	208	100	161.2	162	1.06	0

The elugram of the Sample C1 is presented in Figure 11.

## Supplementary Materials

The following are available online at https://www.mdpi.com/article/10.3390/polym13203505/s1. Figure S1. Predicted 1H NMR specter of possible side product with 8-member ring (as presented on Figure 8). Figure S2. Predicted ^1^H NMR specter of possible side product with 5-member ring (as presented on Figure 9). Figure S3. SEC elugram of sample R1 (a) macroinitiator; (b) diblock-copolymer; (c) UV/RI ratio for sample R1.
