# Supplementary material for: Polystyrene-b-Poly(2-(Methoxyethoxy)ethyl Methacrylate) Polymerization by Different Controlled Polymerization Mechanisms
Source: Polymers (Basel). 2021 Oct 12;13(20):3505. doi: 10.3390/polym13203505 (PMC8540230; doi:10.3390/polym13203505)
Supplement: Supplementary file 1 [file polymers-13-03505-s001.zip › polymers-1382652-supplementary.pdf]

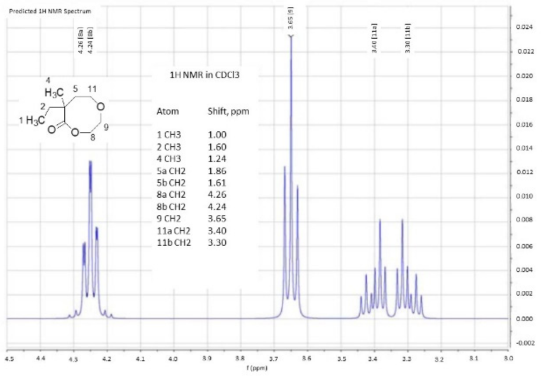

Figure S1. Predicted ^{1}H NMR specter of possible side product with 8-member ring (as presented on Figure 8)

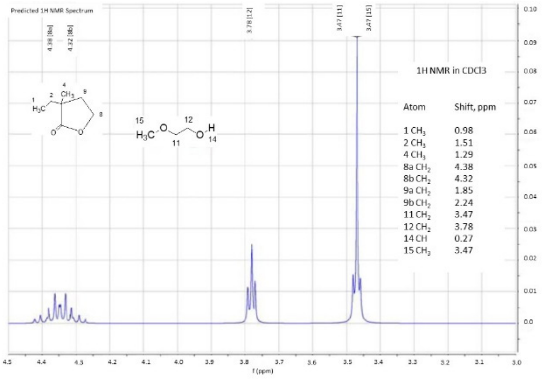

Figure S2. Predicted ^{1}H NMR specter of possible side product with 5-member ring (as presented on Figure 9)

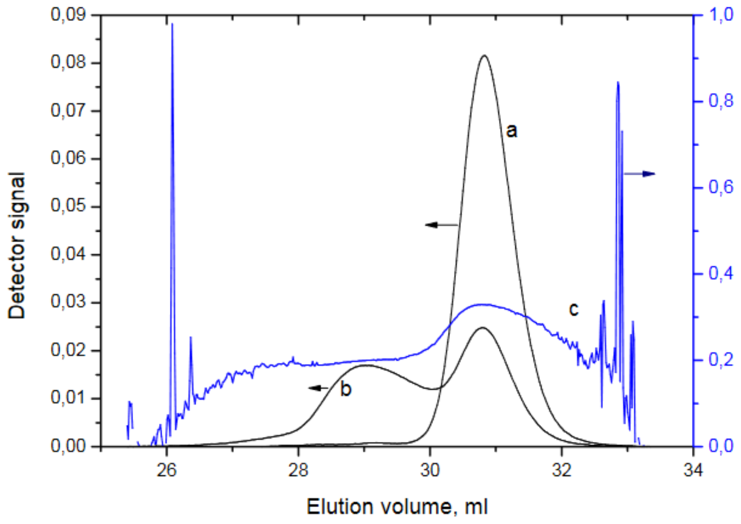

Figure S3. SEC elugram of sample R1 a) macroinitiator; b) diblock-copolymer; c) UV/RI ratio for sample R1
